# Validation and utilization of a *TFE3* break-apart FISH assay for Xp11.2 translocation renal cell carcinoma and alveolar soft part sarcoma

**DOI:** 10.1186/s13000-015-0412-z

**Published:** 2015-09-29

**Authors:** Dinesh Pradhan, Somak Roy, Gabriela Quiroga-Garza, Kathleen Cieply, Alyssa L. Mahaffey, Sheldon Bastacky, Rajiv Dhir, Anil V. Parwani

**Affiliations:** Department of Pathology, University of Pittsburgh Medical Center, 200 Lothrop Street, Pittsburgh, 15213 PA USA; Wexner Medical Center, The Ohio State University, Columbus, 43210 OH USA

**Keywords:** TFE3 FISH, Xp11.2 Translocation Renal Cell Carcinoma, Alveolar Soft Part Sarcoma

## Abstract

**Background:**

Xp11.2 or TFE3 translocation renal cell carcinomas (RCC) and alveolar soft part sarcoma (ASPS) are characterized by chromosome translocations involving the Xp11.2 breakpoint resulting in transcription factor TFE3 gene fusions. The most common translocations documented in TFE3 RCCs are t(X;1) (p11.2;q21) and t(X;17) (p11.2;q25) which leads to fusion of *TFE3* gene on Xp11.2 with *PRCC* or *ASPL* respectively. TFE3 immunohistochemistry (IHC) has been inconsistent over time due to background staining problems in part related to fixation issues. Karyotyping to detect TFE3 gene rearrangement requires typically unavailable fresh tissue. Reverse transcriptase-polymerase chain reaction (RT-PCR) is generally very challenging due to degradation of RNA in archival material. The study objective was to develop and validate a *TFE3* break-apart fluorescence in situ hybridization (FISH) assay to confirm Xp11 translocation RCCs and ASPS.

**Methods:**

Representative sections of formalin-fixed paraffin-embedded tissue blocks were selected in 40 possible cases. Approximately 60 tumor cells were analyzed in the targeted region. The validation of *TFE3* FISH was done with 11 negative and two positive cases. Cut off for a positive result was validated as >7.15 % positive nuclei with any pattern of break-apart signals. FISH evaluation was done blinded of the immunohistochemical or karyotype data.

**Results:**

Three out of forty cases were positive for the *TFE3* break-apart signals by FISH. The negative cases were reported as clear cell RCC with papillary features (10), clear cell RCC with sarcomatoid areas (2), Papillary RCC with clear cell areas (9), Chromophobe RCC (2), RCC, unclassified type (3) and renal medullary carcinoma (1). 3 of the negative cases were consultation cases for renal tumor with unknown histology. Seven negative cases were soft tissue tumor suspicious for ASPS.

**Conclusion:**

Our study validates the utility of *TFE3* break-apart FISH on formalin-fixed paraffin-embedded tissue sections for diagnosis and confirmation of Xp11.2 translocation RCCs and ASPS.

## Background

Xp11.2 or TFE3 translocation renal cell carcinoma (RCC) is one of the new entities added in the 2004 World Health Organization (WHO) classification of renal tumors [1]. They are characterized by translocations involving the TFE3 transcription factor located at Xp11.2 locus. The five known gene fusion partners of TFE3 are papillary 1 renal cell carcinoma (PRCC), alveolar soft part sarcoma locus (ASPL), polypyrimidine tract-binding protein-associated splicing factor (PSF), non-POU domain-containing octamer-binding (NonO, p54nrb), and clathrin heavy chain (CLTC) genes, situated on chromosome loci 1q21 [2–5], 17q25 [6–8], 1p34 [9], Xq12 [9], and 17q23 [10] respectively. The t(X; 17) (p11.2; q25) or TFE3-ASPL translocation in RCC and alveolar soft part sarcoma (ASPS) contain the identical TFE3-ASPL fusion transcript; however, the t(X; 17) translocation is consistently balanced (reciprocal) in the Xp11.2 translocation RCC and unbalanced in the ASPS [11]. In translocation RCC involving t(X; 10) (p11; q23) or t(X;3) (p11.2;q23), the participating gene which fuses to TFE3 remains unknown [12, 13].

Xp11.2 RCCs are classically recognized as pediatric RCC affecting children and young adults [14–20]. These tumors are considered aggressive with early age of onset and variable morphologic features including clear cell or eosinophilic morphology, and papillary or alveolar architecture [21]. This entity seems to be underdiagnosed and misclassified as clear cell or papillary RCC in adults, because of overlapping morphologic features. Definite diagnosis in suspicious cases requires confirmation of the presence of TFE3 protein by immunohistochemistry (IHC) or *TFE3* gene rearrangement by karyotyping or reverse transcriptase-polymerase chain reaction (RT-PCR) to detect chimeric TFE3 mRNA fusion transcripts. TFE3 IHC, though less time consuming and relatively less expensive, has been inconsistent over time due to background staining problems [22]. Besides variable fixation time, especially common in consultation cases, gives variable results [23]. False positive may often be seen due to titration problem. Karyotyping requires fresh tissue which is generally not sent for cytogenetic analysis of adult renal masses in most institutes. RT-PCR on formalin-fixed, paraffin-embedded (FFPE) tissue is infrequently used as a diagnostic tool. It is also very challenging as fresh tissue is rarely available and there is degradation of RNA in the archival material. Moreover, it may necessitate multiple PCRs to cover all the known partners of TFE3.

ASPS is a rare soft tissue tumor which has ASPL-TFE3 gene fusion as a result of unbalanced translocation der (17) t(X;17) (p11;q25) or rarely a balanced translocation t(X;17) (p11;q25). The classical alveolar pattern surrounded by fibrous septa and large round to oval tumor cells is fairly non-specific requiring help from ancillary studies [11].

As the morphology of both Xp11.2 RCCs and ASPS are non-specific and there are a lot of technical difficulties with the available ancillary tools – IHC limited by equivocal results, karyotyping limited by availability of viable tumor cells and RT-PCR limited by RNA quality, we tried to validate and utilize TFE3 break-apart fluorescence in-situ hybridization (FISH) assay in FFPE tissue to confirm the diagnosis of an Xp11.2 RCC and ASPS. Eventually, we find that a break-apart FISH assay is an excellent diagnostic and confirmatory test in the evaluation of TFE3 gene rearrangement in primary as well as metastatic Xp11.2 RCCs and other TFE3 tumors.

## Methods

**Fig. 1:**
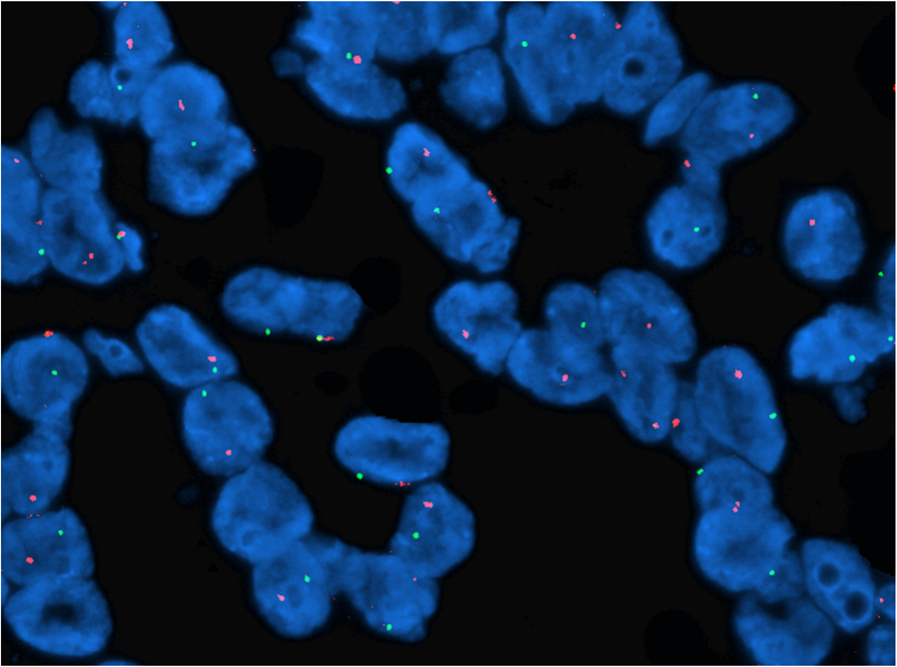
*TFE3* FISH image indicating *TFE3* gene rearrangement with widely separated red and green signals

FFPE tissue blocks were serially sectioned at 4 μ intervals. Hematoxylin and eosin (H&E) sections were used to determine the area of the tissue to be targeted for analysis. FISH slides were deparaffinized in xylene twice for 10 min, dehydrated twice with 100 % ethanol and then pretreated using the Vysis Paraffin Pretreatment Kit (Abbott Molecular, Des Plaines, IL). Slides were digested for 36 min in protease solution (0.5 mg/ml) at 37 °C. TFE3 FISH was performed using a dual-color break apart probe labeled in Texas Red and FITC (Abnova Co., Taipei, Taiwan). The target slide was denatured in 70 % Formamide at 75 °C for 5 min and dehydrated in 70, 85, and 100 % ethanol. Slides were incubated with probe overnight at 42 °C in a humidified chamber. Post-hybridization washes were performed using 2 × SSC/0.3 % Igepal at 73 °C for 2 min (Sigma, St. Louis, MO). Slides were air-dried in the dark and counterstained with 4,6-diamidino-2-phenylindole (DAPI)/antifade (Abbott Molecular). All slides were kept at 4 °C in the dark after hybridization. Analysis was performed using a Leica DM5500 B fluorescence microscope (Leica Microsystems) and CytoVision Workstation (Applied Imaging, Santa Clara, CA) equipped with Chroma Technology 83,000 filter set with single and dual band excitors for Texas Red, Spectrum Green, and DAPI (uv 360 nm) (Abbott Molecular). Only individual and well delineated cells were scored. Overlapping cells were excluded from the analysis. Approximately 60 tumor cell nuclei were analyzed in the targeted region by each of the 2 experienced technicians. The expected normal nuclei had 2 fusion signals reflecting intact TFE3 alleles in a female individual and 1 fusion signal reflecting an intact TFE3 allele in a male individual. The signal pattern 1 red 1 green 1 fusion (yellow) was the most common positive pattern for a balanced TFE3 translocation in a female individual, whereas the signal pattern 1 red 1 green was the most typical positive pattern for a balanced TFE3 rearrangement in a male individual (Fig. 1). Unbalanced translocations in a female individual yielded a 1 red 2 fusion pattern. To be scored as a break apart and to avoid false positive, the signals had to be separated by >2 signal diameters. To avoid false negative in a 4 μ section where red or green signal may be out of the visible plane of section, a minimum of 60 nuclei were evaluated per case.

To determine a cutoff for a positive result, 11 negative and 2 positive cases were evaluated using statistical criteria. Cut off for a positive result was calculated as >7.15 % positive nuclei with any pattern of break-apart signals when 60 nuclei were scored thoroughly. FISH evaluation was done blinded of the IHC or karyotype data.

## Results

A total of 40 cases were evaluated for a TFE3 gene rearrangement using a break-apart FISH which comprised 33 renal neoplasms and 7 soft tissue sarcomas suspicious for Xp11.2 RCC and ASPS respectively. The evaluation was done blinded of morphologic possibility, karyotype, and TFE3 IHC results. Of the 40 cases, 3 cases were positive for the *TFE3* break-apart signals by FISH. On retrospective analysis of these 3 cases, one was a 26-year-old female with unilateral renal mass diagnosed morphologically as chromophobe RCC arising in a cyst, stage pT1b, positive for CD-10, E-cadherin, caveolin and parvalbumin; focally positive for AE1/AE3, CAM5.2, CK7, EMA, RCC antigen and colloidal iron; and negative for carbonic anhydrase IX and BerEP4. This case was later subjected to TFE3 IHC which was positive. The cytogenetic analysis of this case revealed a novel translocation involving t(X;19) (p11.2;q13.1). The other was a renal tumor consultation case in a 10-year-old male child. The third positive case was a 39-year-old male with 12.8 cm retroperitoneal mass diagnosed as pT2b ASPS in 2002. He had a left upper lobe lung metastasis in 2006 and a jejunal metastasis in 2014. This jejunal metastasis revealed the TFE3 gene rearrangement when subjected to break apart FISH. The negative cases were reported as clear cell RCC with papillary features (10), clear cell RCC with sarcomatoid areas (2), Papillary RCC with clear cell areas (9), Chromophobe RCC (2), RCC, unclassified type (3) and renal medullary carcinoma (1). Three of the negative cases were consultation cases for renal tumor with unknown histology. Seven negative cases were soft tissue tumor suspicious for ASPS.

FISH evaluation of tissue used for validation purpose showed break-apart signals in both positive cases, each of TFE3 RCC and ASPS, but in none of the non-Xp11.2 RCCs and normal tissue.

## Discussion

Xp11.2 translocation carcinoma is a recently recognized entity in the 2004 WHO renal tumor classification [1]. It is an uncommon tumor generally arising in children and young adults but recently has been recognized in adults as old as 58 years [24–36]. To date, 5 distinct reciprocal gene fusion partners of the TFE3 gene located at Xp11.2 have been recognized in RCCs which include ASPL, PRCC, PSF, NonO, and CLTC situated on chromosome loci 17q25, 1q21, 1p34, Xq12, and 17q23 respectively [2–10]. Another rare group of renal carcinomas showing the translocation t(6; 11) (p21; q12) involving transcription factor EB (TFEB) has also been reported [37, 38]. TFE3 and TFEB belong to the microphthalmia transcription factor (MiTF) subfamily, which also includes MiTF and transcription factor EC. Argani and Ladanyi have proposed regrouping these neoplasms into the category of MiTF/TFE family translocation carcinomas [21].

The most characteristic morphologic pattern of the Xp11.2 translocation RCC is that of an epithelioid neoplasm with predominance of clear cells, papillary architecture and psammoma bodies. TFE3 translocation RCCs regularly express CD10 and the RCC marker, and most express the renal transcription factors PAX2 and PAX8. In contrast, they are less immunoreactive for epithelial markers such as cytokeratins and EMA [29]. Few of them are reactive for melanocytic markers such as Melan A and HMB45. Infrequently, they may also express papain-like cystein protease cathepsin K. These tumors show characteristically strong nuclear TFE3 immunostaining [39, 40].

ASPS is a rare soft tissue tumor that harbors the ASPL-TFE3 gene fusion. These tumors are most often seen in the deep soft tissues of the extremities. Classically, the tumors show the distinctive alveolar growth pattern and strong nuclear immunostaining for TFE3 [41].

The morphologic differential diagnosis of Xp11.2 translocation RCC is quite broad. The most common renal neoplasms that mimic TFE3 RCCs are those with clear cells and papillary architecture. These include clear cell RCC with focal papillary/pseudopapillary areas, papillary RCC with focal clear cell areas and clear cell papillary RCC. Diffuse carbonic Anhydrase-IX (CA-IX) immune-labeling of clear cell RCC and cytokeratin 7 labeling of papillary RCC may help differentiate the TFE3 RCCs, which will show strong nuclear immunoreactivity for TFE3 IHC. Clear cell papillary RCC typically shows a branching tubular architecture, has apically aligned nuclei with subnuclear clearing, and reveals a low nuclear grade. Other neoplasms that may be confused with Xp11.2 translocation RCC include chromophobe RCC. Diffuse CD117 labeling favors chromophobe RCC over Xp11 translocation RCC.

Besides Xp11 translocation RCC and ASPS, perivascular epithelioid cell tumors (PEComas) have also shown immunoreactivity for TFE3. Folpe et al. showed that five of 17 PEComas were TFE3 positive [42]. Recently, a case of PEComa with PSF-TFE3 gene fusion proven by FISH and RT-PCR has been reported [43]. Interestingly, Argani et al. reported a distinctive type of renal cancer with overlapping features of PEComa, Xp11 translocation carcinoma, and melanoma [44].

The diagnosis of Xp11 translocation carcinoma can be problematic in cases of unusual clinical or morphologic presentation or technical difficulty with TFE3 IHC staining. TFE3 IHC may be inconsistent due to many reasons like variation in staining in different lot because of polyclonal antibody, fixation dependence of the antibody and the subjectivity in interpretation of the TFE3 IHC. As such, cytogenetic examination of fresh tissue remains the gold standard. When fresh tissue is unavailable, FISH and/or RT-PCR can be performed on FFPE tissues. However, RT-PCR of archival tissue is difficult due to degradation of RNA, insufficient extraction efficiency and difficulty with the availability of adequate material in small biopsy samples. In addition, Xp11.2 translocation RCC has at least five known fusion partners with TFE3 making RT-PCR more time consuming and labor intensive. Potential unknown translocation(s) involving the TFE3 gene may yield false negative results. FISH on the other hand represents a cost and time-efficient method that uses FFPE tissue. Aulmann et al. has reported the feasibility of detecting the ASPS-TFE3 gene fusion in ASPS with both split and fusion probes [45]. Their paper is the first development of a FISH assay for the detection of TFE3 gene translocation in paraffin-embedded tissues. The advantage of the break apart probe type FISH assay is that though the fusion partner with TFE3 is not identified, but potentially all translocations involving TFE3 can be detected. Because all the probes target the X chromosome, the patient’s sex is important for FISH interpretation. The male patient should have one pair of signals (1 X chromosome) and the female patient should have two pairs of signals (2 X chromosomes).

## Conclusion

In this study, we validated a TFE3 break apart FISH assay to aid as a relatively rapid test for detecting Xp11.2 translocation in cases of TFE3 RCC and ASPS. This FISH assay can be utilized as an adjunct to morphology and immunohistochemistry to diagnose TFE3-associated carcinomas and other neoplasms.
